# The Fool-Killer

**Published:** 1875-02

**Authors:** 


THE FOOL-KILLER.—This personage is still actively employed, perhaps never more actively than now. He works in various ways and with numerous potent weapons, but with none more potent than tobacco. Within a week past a young man, of 28 years, died in New York of heart disease, induced solely by smoking. He had smoked but 10 years, and not generally to excess—as he understood the term—but he had what the doctors call "a tobacco heart"—that is to say, a heart which, through the poisonous herb, had ceased its normal action, the regularity and rythm of which, were "Like sweet bells jangled, out of tune;" and so his useful life was cut short. He only smoked three or four cigars a day. To be sure he would frequently smoke a couple of strong ones in the evening, and some days he would smoke nearly all the time, if his surroundings favored it. But he was sure he "didn't smoke to excess," and in this conviction he died at twenty-eight.

Another young man, of twenty-five, was so strong and well that he could smoke ten or twenty cigars a day, and not feel at all the worse for it. He was positive that smoking never would hurt him, though it might injure some weak constitutions. He laid a wager that he would smoke twelve cigars in two hours. He went at it with all the conscious power of a man who knew his business, and was equal to it. At the end of the ninth cigar he grew dizzy, the symptoms becoming more decided until the twelve were smoked. and the wager won. The bet was a box of cigars. But upon some other fool was the duty of consuming those cigars devolved. The young man was attacked with vomiting, which continued till he died. His few years of smoking had partially poisoned him; his final effort completely paralyzed the heart's action. Yet there never was a time when this young man was willing to admit that he had smoked "to excess."

The truth is, no human being ever used tobacco, either by burning it, and drawing the smoke into mouth, so that the mucus membrane should absorb its poison, or by chewing it, or snuffing it, for a like purpose—who did not use it to excess. One pipe, one cigar, one quid, one pinch of snuff—is an excessive dose, and will be as long as the world stands. It is a poison, and one of the most potent ones known to science. It can be used by some for years, without destroying life, and so can all other poisons. But in nine cases out of ten it kills at last. It is a filthy habit, too, and the wonder is that tobacco users are allowed to live in houses, and to associate with their fellow-beings. We believe the time will come when the consumer of tobacco will be utterly banished from all respectable society, so offensive and repugnant is he to all refined and cleanly persons. This day is coming, surely if slowly. In London to-day, no man who respects himself or seeks the respect of his fellows, is ever seen to smoke in a public place. This sin he perpetrates only at home, among those who are expected to admire or endure him, even though he is a boor and a dirty dog.

---

GASTRALGIA.—The favorite treatment in France for this disease is to apply a poultice of ice for ten minutes, morning and evening, to the pit of the stomach ; second, a mustard plaster to the same spot immediately on removing the ice poultice, to be kept on as long as posible; third, pounded ice, to be taking morning and evening, a tablespoonful every five minutes for an hour; and fourth, a mustard bath three times a week. According to this method the ice of the poultice is to be chopped up into small bits and inclosed in an india rubber bag. The ice taken internally must be pounded with sugar, and must be swallowed down suddenly—not kept in the mouth, as it would thus lose its coldness ; if a tablespoonful prove too much at a time, several teaspoonfuls may be taken instead. The mustard bath is regarded as a powerful tonic.

